# Mothers who have given birth at an advanced age - health status before and after childbirth

**DOI:** 10.1038/s41598-020-66774-4

**Published:** 2020-06-16

**Authors:** Malin Lindell Pettersson, Elizabeth Nedstrand, Marie Bladh, Agneta Skoog Svanberg, Claudia Lampic, Gunilla Sydsjö

**Affiliations:** 1 Department of Obstetrics and Gynecology, and Department of Clinical and Experimental Medicine, Linköping University, Linköping, Sweden; 2 Department of Women’s and Children’s Health, Uppsala University, Uppsala, Sweden; 3 Department of Women’s and Children’s Health, Karolinska Institutet, SE-171 77 Stockholm, Sweden; 4 Department of Public health and Caring Sciences, Uppsala University, SE-751 22 Uppsala, Sweden

**Keywords:** Diseases, Medical research

## Abstract

Women postpone childbirth to an age when morbidity is higher and fertility has decreased and yet the knowledge of mothers’ morbidity related to age remains scarce. Swedish national register data from the Medical Birth Register and National Patient Register was used to investigate the incidence of diseases listed in the International Classification of Diseases, version 10 (ICD-10) in women who gave birth 2007–8. The index group consisted of women 40 years of age or older (n = 8 203) were compared to a control group of women, younger than 40 years (n = 15 569) at childbirth. The period studied was five years before childbirth to five years after. The main outcome measures were incidence of disease diagnosed in specialized hospital care. Demographical data and use of assisted reproduction (ART) were adjusted for. The results showed that older women were more likely to be single; less frequently used tobacco; were educated on a higher level; had a higher BMI and more often had used ART to become pregnant. The older women showed a higher morbidity rate. In the diagnostic groups: Neoplasms, Blood and immune system, Eye and adnexa, Ear and mastoid, Circulatory, Digestive, Skin and subcutaneous tissue, Musculoskeletal and connective tissue, and Genitourinary. The results add to the body of knowledge of a number of specific risks faced by older mothers and may be used to identify preventive actions concerning fertility and morbidity both before and after childbirth.

## Introduction

During the last decades, postponing childbearing has increased significantly among women^1–3^ in developed countries. In the three Nordic countries, Denmark, Norway, and Sweden, the mean age at first delivery increased from 23,5–24 years in 1975 to 29–29,5 years in 2018^4–7^ and the trend has been similar in most developed countries^3^. In Sweden in 1982, only 304 children were born to women 40 years or older but by 2016 women in this age group gave birth to 1083 children. Only 42 children were born to women 45 years and older in Sweden in 1982 while in 2016 almost a four-fold increase is seen, 203 children^8^. This increase in number of children born to women at or above the age of 40 might be a consequence of the growing importance of postponing childbearing, and of the progress and availability of assisted reproductive technology (ART)^3,9^.

Advanced maternal age at childbirth is associated with a higher level of morbidity^10^ and studies have shown that older mothers, already by the age of 35, exhibit a higher morbidity and consume more inpatient care, both before and after pregnancy than their younger counterparts^11^. First-time mothers age 35 or older had significantly more days of sick leave two years before and up to one year after childbirth compared to younger mothers in a population-based cohort study by Brehmer *et al*.^11^. Norwegian researchers have reported that primiparous mothers at an advanced age (≥32 years of age) and very advanced age (≥38 years of age) were more likely to experience psychological distress and lower satisfaction with life during pregnancy and up to three years after childbirth compared to mothers age 25–31^12,13^.

Mental health problems are common among women in the reproductive period. Women who give birth run three times the risk of being diagnosed with a psychiatric disorder postpartum compared to women not giving birth^14^. Having previous experience of psychiatric illness is a well-known risk factor for affective disorders during pregnancy and postpartum^11,15–18^. Pregnancy and childbirth can also trigger the debut of several psychiatric disorders^14^.

Recent studies conclude that single status of pregnant women is a socio-demographic factor highly correlated with advanced maternal age^19–21^. In research on parental health and children’s wellbeing, being a single mother often constitutes a risk factor that tends to have negative effects on the health of mother^22–25^. Already at childbirth the mother’s single status is associated with adverse perinatal outcomes^21,26^. Previous research on single-mother households seldom distinguished single mothers who were said to be “single by choice” or referred to as “solo mothers” from those who were single due to divorce, death of a spouse or partner, or unplanned pregnancy.

Childbirth clearly constitutes a risk factor for the development of psychiatric problems in women, and maternal age seems to be a mediating variable for both psychiatric and physical morbidity. However, the magnitude of the association between childbirth and physical morbidity has not yet been sufficiently investigated.

Thus, the aim of the present study was to study morbidity in a national sample of mothers 40 years of age or older before and after childbirth, and compare them with mothers under the age of 40. An additional aim was to investigate whether morbidity before and after childbirth co-varies with marital status and with the use of ART.

## Method

The data was retrieved from a study that was designed as case-control study where mothers who had given birth in Sweden between 2007 and 2012 age 40 years or older formed the index group, n = 37 558 that was then compared to a control group of mothers under the age of 40, n = 7172. For the purpose of the present study, mothers who had given birth during 2007–2008 were selected to allow for a follow-up of up to 5 years after giving birth. The study population consisted of 23 772 mothers; 8203 (34%) mothers age 40 years or older in the index group and 15 569 (66%) in the control group.

### Registers

The data used in this study was retrieved from The Swedish Medical Birth Register (MBR) and The National Patient Register (NPR). The Swedish National Board of Health and Welfare maintains both registers. The MBR contains medical information on practically all deliveries in Sweden from 1973 until the present^27^ and has been found to be of good quality^28^. The NPR contains information pertaining to hospital care, inpatient data since 1964, and specialized outpatient care data since 2001. At the beginning, the NPR was focused on psychiatric diagnoses, but since 1987 all inpatient visits have been recorded in the NPR. NPR has been evaluated and deemed to be of good quality^29,30^.

### Definitions

Maternal age when giving birth was categorized into two levels, “≤39 years of age”, “≥40 years of age”, civil status was categorized into “Married/cohabiting” and “Single” while educational level was defined as “Elementary”, “High school”, and “College” as registered at the time of childbirth. Further, tobacco use during pregnancy, twinning, chronic disease, and ART were each categorized into “No” and “Yes”, mother’s BMI at registration was divided into “Normal”, “Underweight”, and “Overweight/obese” while parity was categorized into “Primiparous” and “Multiparous”.

Morbidity up to 5 years prior to childbirth as well as up to 5 years after childbirth was investigated by the number of incidence of diagnoses within each of the first 14 main chapters defined in the International Classification of Diseases, version 10 (ICD-10). For each chapter, indicator variables were created where the levels indicated either “No chapter specific diagnosis during follow-up” or “Having at least one chapter specific diagnosis during follow-up”, these were retrieved by type of visit (in- or outpatient) and summarized into an overall indicator variable for each of the ICD-10 chapters included in the study. In addition, all somatic diagnoses (chapters I-XIV, excluding chapter V) were summarized into a total indicator; Any type of somatic disease, where the two levels were “No somatic diagnosis during the follow-up” and “At least one somatic diagnosis during the follow-up”.

### Diagnoses

*Certain infections and parasitic diseases* (Chapter I, codes A00-B99), *Neoplasms* (Chapter II, codes C00-D48), *Diseases of the blood and blood-forming organs and certain disorders involving the immune mechanism* (Chapter III, codes D50-D89), *Endocrine, nutritional and metabolic diseases* (Chapter IV, codes E00-E90), *Mental and behavioural disorders* (Chapter V, codes F00-F99), *Diseases of the nervous system* (Chapter VI, codes G00-G99), *Diseases of the eye and adnexa* (Chapter VII, codes H00-H59), *Diseases of the ear and mastoid process* (Chapter VIII, codes H60-H95), *Diseases of the circulatory system* (Chapter IX, codes I00-I99), *Diseases of the respiratory system* (Chapter X, codes J00-J99), *Diseases of the digestive system* (Chapter XI, codes K00-K93), *Diseases of the skin and subcutaneous tissue* (Chapter XII, codes L00-L99), *Diseases of the musculoskeletal and connective tissue* (Chapter XIII, codes M00-M99), and *Diseases of the genitourinary system* (Chapter XIV, codes N00-N99).

### Statistical analysis

The relationship between age when becoming a mother and socio-demographic and medical data, as well as morbidity, was initially analyzed using Pearson’s Chi-square statistic. Multivariable analyses included multiple logistic regression models to calculate the odds ratio of being diagnosed with chapter-specific diagnoses, each modeled separately. Dependent variables were the indicator variables for having a chapter-specific diagnosis and independent variables included were: Age when giving birth, Civil status, ART treatment, Educational level, Tobacco use during pregnancy, Mother’s BMI at registration, Parity, Twin birth, and Chronic disease, and for the analyses of morbidity up to 5 years after delivery the indicator variable “Any somatic disease” regarding morbidity up to 5 years prior to delivery was also included. To further investigate the relationship between age and morbidity, the same set of multiple regression models were performed on age-stratified data to evaluate age specific risk factors. A p-value < 0.05 (two-sided) was considered statistically significant. All analyses were performed using SPSS, version 22.0 (IBM Inc., Armonk, NY).

### Ethics approval and consent to participate

This study was approved by the Regional Ethical Review Board, Linköping, Sweden and was performed according to the Declaration of Helsinki. No 2014/111-31. Date 26-03-2016. Informed consent is not applicable. Informed consent was not required, according to national guidelines, since this is a register study with an ethical approval from the ethical review board and permission to use data was obtained from the register holders.

## Results

### Socio-demographic and medical background

By stratifying the data according to type of pregnancy (ART or spontaneous pregnancy) it was found that among mothers who had had a spontaneous pregnancy, women age 40 or older were single to a greater extent, had a higher level of education, and a higher BMI than the younger mothers, Table 1.

**Table 1:** Sociodemographic and medical background data on the study population, reported by age and type of pregnancy.

	Spontaneous pregnancy			ART				
	Mothers ≤ 39	Mothers ≥ 40		Mothers ≤ 39	Mothers ≥ 40		≤39*^a^	≥40*^b^
	n (%)	n (%)	p-value	n (%)	n (%)	p-value	p-value	p-value
**Civil status**			<0.001			0.010	<0.001	<0.001
Married/ cohabiting	13415 (88.9)	6210 (83.0)		462 (97.5)	680 (94.3)			
Single status at registration	1680 (11.1)	1272 (17.0)		12 (2.5)	41 (5.7)			
**Educational level**			<0.001			<0.001	<0.001	<0.001
Elementary	1926 (12.9)	653 (12.9)		26 (5.5)	22 (3.1)			
High school	6080 (40.7)	2791 (37.5)		188 (39.7)	219 (30.4)			
College	6922 (46.4)	4008 (53.8)		260 (54.9)	480 (66.6)			
**Tobacco use during pregnancy**			0.001			0.783	<0.001	<0.001
No	12761 (89.3)	6302 (90.7)		452 (95.4)	689 (95.7)			
Yes	1533 (10.7)	643 (9.3)		22 (4.6)	31 (4.3)			
**Mother’s BMI (registration)**			<0.001			0.060	0.151	0.050
Underweight	236 (1.8)	75 (1.2)		13 (2.9)	7 (1.0)			
Normal	7436 (55.4)	3293 (51.1)		255 (56.7)	379 (56.1)			
Overweight + Obese	5743 (42.8)	3073 (47.7)		182 (40.4)	290 (42.9)			
**Parity**			<0.001			0.380	<0.001	<0.001
Primi	3188 (21.1)	1405 (18.8)		218 (46.0)	313 (43.4)			
Multi	11907 (78.9)	6077 (81.2)		256 (54.0)	408 (56.6)			
**Twin birth**			0.015			0.001	<0.001	<0.001
No	14573 (96.5)	7269 (97.2)		362 (76.4)	607 (84.2)			
Yes	522 (3.5)	213 (2.8)		112 (23.6)	114 (15.8)			
**Chronic disease**			0.151			0.034	0.972	<0.001
No	11996 (79.5)	6007 (80.3)		377 (79.5)	535 (74.2)			
Yes	3099 (20.5)	1475 (19.7)		97 (20.5)	186 (25.8)			

*^a^Spontaneously pregnant women aged 39 years or younger compared to women pregnant with ART.

*^b^Spontaneously pregnant women aged 40 years or older compared to women pregnant with ART.

As for spontaneously pregnant women, women 40 years or older who became pregnant by using ART were more often single, and had a higher level of education, and were less likely to have delivered twins. But in contrast to spontaneously pregnant women, no difference was detected in their BMI, Table 1.

In addition, by stratifying the data according to age it was found that mothers in both age groups who had become pregnant using ART were more often married, had a higher level of education, used tobacco to a lesser extent, were more often primipara, and more often had twins compared to mothers who had become spontaneously pregnant. It was also noted that mothers age 40 years or older and who had been treated with ART more often had a chronic disease, Table 1.

### Morbidity up to 5 years prior to childbirth

In analyzing morbidity up to 5 years prior to childbirth it was found that mothers age 40 years or older who had become spontaneously pregnant were more likely to have been given diagnoses listed in the following chapters *(“Neoplasms”, “Blood and Immune System”, “Endocrine, Nutritional and Metabolic”, “Mental and Behavioral Disorders”, “Nervous System”, “Eye and Adnexa”, “Ear and Mastoid”, “Circulatory”, “Musculoskeletal and Connective Tissue”, and “Genitourinary”)* compared to the mothers in the control group (≤39 years of age), Table 2. On the other hand, spontaneously pregnant mothers in the control group were more likely to have been diagnosed with *“Infections”* and *“Respiratory disease”* compared to mothers age 40 years or older, Table 2.

**Table 2:** Morbidity up to 5 years before childbirth.

	Spontaneous pregnancy			ART				
	Mothers ≤ 39	Mothers ≥ 40		Mothers ≤ 39	Mothers ≥ 40		≤39*^a^	≥40*^b^
	n (%)	n (%)	p-value	n (%)	n (%)	p-value	p-value	p-value
**Infections (Chapter I)**			0.002			0.656	0.732	0.361
No	13731 (91.0)	6900 (92.2)		429 (90.5)	658 (91.3)			
Yes	1364 (9.0)	582 (7.8)		45 (9.5)	63 (8.7)			
**Neoplasms (Chapter II)**			<0.001			0.028	0.026	0.001
No	14061 (93.2)	6749 (90.2)		429 (90.5)	622 (86.3)			
Yes	1034 (6.8)	733 (9.8)		45 (9.5)	99 (13.7)			
**Blood and Immune system (Chapter III)**	0.005			0.974	0.264	0.771		
No	14782 (98.0)	7288 (97.4)		461 (97.3)	701 (97.2)			
Yes	303 (2.0)	194 (2.6)		13 (2.7)	20 (2.8)			
**Endocrine, nutritional and metabolic (Chapter IV)**	<0.001			0.272	<0.001	<0.001		
No	14287 (94.6)	6984 (93.3)		410 (86.5)	639 (88.6)			
Yes	808 (5.4)	498 (6.7)		64 (13.5)	82 (11.4)			
**Mental and behavioral disorders (Chapter V)**		0.018			0.718	0.382	0.147	
No	14013 (92.8)	6880 (92.0)		445 (93.9)	674 (93.5)			
Yes	1082 (7.2)	602 (8.0)		29 (6.1)	47 (6.5)			
**Nervous system (Chapter VI)**			0.016			0.932	0.201	0.029
No	14397 (95.4)	7081 (94.6)		458 (96.6)	696 (96.5)			
Yes	698 (4.6)	698 (5.4)		16 (3.4)	25 (3.5)			
**Eye and adnexa (Chapter VII)**			0.012			0.913	0.276	0.606
No	14511 (96.1)	7140 (95.4)		451 (95.1)	685 (95.0)			
Yes	584 (3.9)	342 (4.6)		23 (4.9)	36 (5.0)			
**Ear and mastoid (Chapter VIII)**			0.001			0.023	0.040	0.691
No	14710 (97.4)	7233 (96.7)		469 (98.9)	699 (96.9)			
Yes	385 (2.6)	249 (3.3)		5 (1.1)	22 (3.1)			
**Circulatory (Chapter IX)**			<0.001			0.110	0.212	0.213
No	14587 (96.6)	7102 (94.9)		463 (97.7)	692 (96.0)			
Yes	508 (3.4)	380 (5.1)		11 (2.3)	29 (4.0)			
**Respiratory (Chapter X)**			0.009			0.980	0.837	0.227
No	13955 (92.4)	6989 (93.4)		437 (92.2)	665 (92.2)			
Yes	1140 (7.6)	493 (6.6)		37 (7.8)	56 (7.8)			
**Digestive (Chapter XI)**			0.410			0.811	0.763	0.672
No	13631 (90.3)	6782 (90.6)		430 (90.7)	657 (91.1)			
Yes	1464 (9.7)	700 (9.4)		44 (9.3)	64 (8.9)			
**Skin and subcutaneous tissue (Chapter XII)**		0.924			0.720	0.130	0.237	
No	13888 (92.0)	6881 (92.0)		427 (90.1)	654 (90.7)			
Yes	1207 (8.0)	601 (8.0)		47 (9.9)	67 (9.3)			
**Musculoskeletal and connective tissue (Chapter XIII)**	<0.001			0.366	0.962	0.491		
No	13716 (90.9)	6619 (88.5)		431 (90.9)	644 (89.3)			
Yes	1379 (9.1)	863 (11.5)		43 (9.1)	77 (10.7)			
**Genitourinary (Chapter XIV)**			0.002			<0.001	<0.001	<0.001
No	11247 (74.5)	5430 (72.6)		140 (29.5)	314 (43.6)			
Yes	3848 (25.5)	2052 (27.4)		334 (70.5)	407 (56.4)			
Somatic disease*^c^			<0.001			<0.001	<0.001	<0.001
No	6935 (45.9)	3121 (41.7)		76 (16.0)	175 (24.3)			
Yes	8160 (54.1)	4361 (58.3)		398 (84.0)	546 (75.7)			

*^a^Spontaneously pregnant women age 39 years or younger compared to women pregnant with ART.

*^b^Spontaneously pregnant women age 40 years or older compared to women pregnant with ART.

*^c^Chapters 1 + 2 + 3 + 4 + 6 + 7 + 8 + 9 + 10 + 11 + 12 + 13 + 14.

The only statistically significant differences regarding morbidity in relation to the mother’s age among mothers who had undergone ART treatment to achieve pregnancy were for the diagnoses *“Neoplasms”* and *“Ear and Mastoid”* where older mothers were more likely to be given these diagnoses compared to younger mothers. Younger mothers were more likely to have been diagnosed with genitourinary disease, Table 2. By stratifying by age, it was found that women in both groups who had been treated with ART were more likely to have been diagnosed with *“Neoplasms”* and *“Endocrine, nutritional and metabolic disease”* compared to mothers who had become spontaneously pregnant. Mothers of all ages who had been treated with ART were more likely to have been diagnosed with a *“Genitourinary disease”* and *“Any type of somatic disease”* compared to women with a spontaneous pregnancy, Table 2.

### Morbidity up to 5 years after childbirth

In Table 3, data on mothers’ morbidity up to five years after childbirth are displayed. Older mothers who had become spontaneously pregnant were more likely to have been diagnosed with these conditions: (*“Neoplasms”, Nervous System”, “Eye and Adnexa”, “Ear and Mastoid”, “Circulatory”, “Skin and Subcutaneous Tissue”, “Musculoskeletal and Connective Tissue”, “Genitourinary”*) as well as *“Any type of somatic disease”* compared to the younger age group (≤39 years of age). Amongst the mothers who had used ART to become pregnant, the older mothers had more often been given the diagnosis *“Neoplasms”* and *“Eye and Adnexa”* than the younger mothers had. Younger mothers, pregnant with ART, on the other hand, had more often been diagnosed with *“Genitourinary disorder”* as well as *“Any type of somatic disease”*, Table 3.

**Table 3:** Morbidity up to 5 years after childbirth.

	Spontaneous pregnancy			ART				
	Mothers ≤ 39	Mothers ≥ 40		Mothers ≤ 39	Mothers ≥ 40		≤39*^a^	≥40*^b^
	n (%)	n (%)	p-value	n (%)	n (%)	p-value	p-value	p-value
**Infections (Chapter I)**			0.147			0.330	0.956	0.028
No	13832 (91.6)	6898 (92.2)		434 (91.6)	648 (89.9)			
Yes	1263 (8.4)	584 (7.8)		40 (8.4)	73 (10.1)			
**Neoplasms (Chapter II)**			<0.001			0.030	0.126	0.005
No	13544 (89.7)	6485 (86.6)		415 (87.6)	598 (82.9)			
Yes	1551 (10.3)	997 (13.3)		59 (12.4)	123 (17.1)			
**Blood and Immune system (Chapter III)**		0.913			0.087	0.363	0.112	
No	14635 (97.0)	7252 (96.9)		463 (97.7)	691 (95.8)			
Yes	460 (3.0)	230 (3.1)		11 (2.3)	30 (4.2)			
**Endocrine, nutritional and metabolic (Chapter IV)**		0.179			0.678	0.044	0.025	
No	13637 (90.3)	6801 (90.9)		415 (87.6)	637 (88.3)			
Yes	1458 (9.7)	681 (9.1)		59 (12.4)	84 (11.7)			
**Mental and behavioral disorders (Chapter V)**		0.448			0.918	0.300	0.404	
No	13672 (90.6)	6800 (90.9)		436 (92.0)	662 (91.8)			
Yes	1423 (9.4)	682 (9.1)		38 (8.0)	59 (8.2)			
**Nervous system (Chapter VI)**			0.045			0.616	0.294	0.196
No	14159 (93.8)	6966 (93.1)		439 (92.6)	662 (91.8)			
Yes	936 (6.2)	516 (6.9)		35 (7.4)	59 (8.2)			
**Eye and adnexa (Chapter VII)**			<0.001			0.015	0.061	<0.001
No	14198 (94.1)	6855 (91.6)		436 (92.0)	631 (87.5)			
Yes	897 (5.9)	627 (8.4)		38 (8.0)	90 (12.5)			
**Ear and mastoid (Chapter VIII)**			<0.001			0.385	0.866	0.933
No	14544 (96.3)	7124 (95.2)		456 (96.2)	686 (95.1)			
Yes	551 (3.7)	358 (4.8)		18 (3.8)	35 (4.9)			
**Circulatory (Chapter IX)**			<0.001			0.196	0.708	0.463
No	14172 (93.9)	6852 (91.6)		447 (93.9)	666 (92.4)			
Yes	923 (6.1)	630 (8.4)		27 (5.7)	55 (7.6)			
**Respiratory (Chapter X)**			0.948			0.160	0.386	0.001
No	13711 (90.8)	6798 (90.9)		425 (89.7)	627 (87.0)			
Yes	1384 (9.2)	684 (9.1)		49 (10.3)	94 (13.0)			
**Digestive (Chapter XI)**			0.058			0.929	0.743	0.957
No	13291 (88.0)	6522 (87.2)		415 (87.6)	629 (87.2)			
Yes	1804 (12.0)	960 (12.8)		59 (12.4)	92 (12.8)			
**Skin and subcutaneous tissue (Chapter XII)**			0.009			0.244	0.202	0.016
No	13582 (90.0)	6648 (88.9)		418 (88.2)	619 (85.9)			
Yes	1513 (10.0)	834 (11.1)		56 (11.8)	102 (14.1)			
**Musculoskeletal and connective tissue (Chapter XIII)**		<0.001			0.366	0.962	0.491	
No	13716 (90.9)	6619 (88.5)		431 (90.9)	644 (89.3)			
Yes	1379 (9.1)	863 (11.5)		43 (9.1)	77 (10.7)			
**Genitourinary (Chapter XIV)**			<0.001			<0.001	<0.001	<0.001
No	11093 (73.5)	5314 (71.0)		141 (29.7)	310 (43.0)			
Yes	4002 (26.5)	2168 (29.0)		333 (70.3)	411 (57.0)			
Somatic disease*			<0.001			<0.001	<0.001	<0.001
No	5662 (37.5)	2509 (33.5)		65 (13.7)	111 (15.4)			
Yes	9433 (62.5)	4973 (66.4)		409 (86.3)	610 (84.6)			

*^a^Spontaneously pregnant women age 39 years or younger compared to women pregnant with ART.

*^b^Spontaneously pregnant women age 40 years or older compared to women pregnant with ART.

By stratifying the data by age it was found that mothers 40 years or older who had been treated with ART were more likely to have been diagnosed with *“Infections”, Neoplasms”, “Endocrine, Nutritional and Metabolic”, “Eye and Adnexa, “Respiratory”, “Genitourinary”*, and *“Any type of somatic disease”* after childbirth, compared to mothers age 40 years or older who had become spontaneously pregnant, Table 3. Moreover, mothers in the control group who had been treated with ART were more likely to have been given the diagnoses *“Endocrine, Nutritional and Metabolic”, “Genitourinary”* and *“Any type of somatic disease”* compared to the control-group mothers who were under 40 years of age and had become spontaneously pregnant, Table 3.

### Descriptive analysis of morbidity up to 5 years prior to and after childbirth

To further investigate the relationship between morbidity and single status, usage of ART, and age when giving birth, multiple logistic regression was performed for each of the ICD-10 chapter diagnoses. Dependent variables were the indicator for having a chapter-specific diagnosis, and all the variables presented in Table 1 were included as independent variables (Civil status, Educational level, Tobacco use during pregnancy, Mother’s BMI at registration, Parity, Twin birth, and Chronic disease), and for the analyses of morbidity up to 5 years prior and 5 years after delivery the indicator variable *“Any type of somatic disease”* was included.

### Multivariable analysis of morbidity up to 5 years prior to childbirth

In the analyses of morbidity up to 5 years prior to childbirth it was found that mothers who were single, had used ART to achieve pregnancy, and age 40 years or older when giving birth were more likely to have been diagnosed with *“Any type of somatic disease”* than mothers who did not have any of these attributes (Single: OR = 1.19, 95% CI = 1.06–1.33; ART usage: OR = 3.18, 95% CI = 2.73–3.70; Age ≥40: OR = 1.15, 95% CI = 1.09–1.22), Table 4. In addition, being single increased the odds ratio for being given the diagnoses *“Infections”* (OR = 1.48, 95% CI = 1.26–1.74), *“Blood and immune system”* (OR = 1.46, 95% = 1.07–1.99), *“Mental and behavioral disorders”* (OR = 1.85, 95% CI = 1.58–2.16), and *“Respiratory”* (OR = 1.25, 95% CI = 1.03–1.52), Table 4. Having used ART, compared to becoming spontaneously pregnant, increased the likelihood of having been diagnosed with *“Genitourinary disorders”* (OR = 4.85, 95% = 4.25–5.53), “*Neoplasms* (OR = 1.42 95% = 1.16–1.72), *“Endocrine, nutritional and metabolic”* (OR = 2.23 95% = 1.81–2.74) up to 5 years prior to childbirth. Considering the importance of age on morbidity up to 5 years prior to childbirth it was noted that mothers in the index group had a reduced likelihood of having been diagnosed with *“Infections”* and *“Respiratory”* compared to mothers in the control group. In addition, mothers in the index group had an increased likelihood of being diagnosed with *“Neoplasms”* (OR = 1.43, 95% CI = 1.29–1.59), *“Blood and immune system”* (OR = 1.31, 95% CI = 1.08–1.59), *“Endocrine, Nutritional and Metabolic”* (OR = 1.19, 95% CI = 1.06–1.35), *“Mental and Behavioral Disorders”* (OR = 1.25, 95% CI = 1.12–1.39), *“Ear and mastoid”* (OR = 1.36, 95% CI = 1.14–1.62), *“Circulatory*” (OR = 1.55, 95% CI = 1.34–1.79), *“Musculoskeletal and connective tissue”* (OR = 1.29, 95% CI = 1.18–1.42), and *“Any type of somatic disease”* (OR = 1.15, 95% CI = 1.09–1.22) compared to mothers in the control group, Table 4.

**Table 4:** Odds ratios and corresponding 95% confidence intervals (CI) for being diagnosed within each of the presented ICD-10 chapters, up to 5 years before childbirth.

			Up to 5 years before childbirth	
		5 years before childbirth	≤39	≥40
		OR (95% CI)	OR (95% CI)	OR (95% CI)
**Infections (Chapter I)**				
Civil status	Married	*Reference*	*Reference*	*Reference*
	Single	1.48 (1.26–1.74)	1.64 (1.33–2.02)	1.28 (0.98–1.68)
ART	No	*Reference*	*Reference*	*Reference*
	Yes	1.19 (0.96–1.49)	1.12 (0.81–1.56)	1.30 (0.97–1.76)
Age	**≤**39	*Reference*		
	**≥**40	0.85 (0.76–0.95)		
**Neoplasms (Chapter II)**				
Civil status	Married	*Reference*	*Reference*	*Reference*
	Single	1.09 (0.90–1.32)	0.83 (0.60–1.15)	1.28 (1.00–1.62)
ART	No	*Reference*	*Reference*	*Reference*
	Yes	1.42 (1.16–1.72)	1.48 (1.06–2.05)	1.35 (1.06–1.73)
Age	**≤**39	*Reference*		
	**≥**40	1.43 (1.29–1.59)		
**Blood and Immune system (Chapter III)**				
Civil status	Married	*Reference*	*Reference*	*Reference*
	Single	1.46 (1.07–1.99)	1.38 (0.88–2.15)	1.48 (0.96–2.30)
ART	No	*Reference*	*Reference*	*Reference*
	Yes	1.25 (0.84–1.84)	1.76 (0.98–3.18)	0.92 (0.55–1.56)
Age	**≤**39	*Reference*		
	**≥**40	1.31 (1.08–1.59)		
**Endocrine, nutritional and metabolic (Chapter IV)**				
Civil status	Married	*Reference*	*Reference*	*Reference*
	Single	0.87 (0.70–1.09)	0.94 (0.69–1.28)	0.81 (0.58–1.12)
ART	No	*Reference*	*Reference*	*Reference*
	Yes	2.23 (1.81–2.74)	2.97 (2.20–4.03)	1.79 (1.36–2.37)
Age	**≤**39	*Reference*		
	**≥**40	1.19 (1.06–1.35)		
**Mental and behavioral disorders (Chapter V)**				
Civil status	Married	*Reference*	*Reference*	*Reference*
	Single	1.85 (1.58–2.16)	1.84 (1.49–2.28)	1.87 (1.47–2.37)
ART	No	*Reference*	*Reference*	*Reference*
	Yes	0.90 (0.70–1.16)	0.96 (0.64–1.44)	0.87 (0.63–1.21)
Age	**≤**39	*Reference*		
	**≥**40	1.25 (1.12–1.39)		
**Nervous system (Chapter VI)**				
Civil status	Married	*Reference*	*Reference*	*Reference*
	Single	1.08 (0.86–1.37)	1.08 (0.78–1.48)	1.08 (0.77–1.52)
ART	No	*Reference*	*Reference*	*Reference*
	Yes	0.75 (0.53–1.05)	0.86 (0.51–1.47)	0.67 (0.43–1.04)
Age	**≤**39	*Reference*		
	**≥**40	1.12 (0.98–1.28)		
**Eye and adnexa (Chapter VII)**				
Civil status	Married	*Reference*	*Reference*	*Reference*
	Single	1.22 (0.95–1.55)	1.13 (0.79–1.60)	1.32 (0.94–1.86)
ART	No	*Reference*	*Reference*	*Reference*
	Yes	1.25 (0.93–1.66)	1.37 (0.88–2.15)	1.17 (0.80–1.71)
Age	**≤**39	*Reference*		
	**≥**40	1.15 (1.00–1.33)		
**Ear and mastoid (Chapter VIII)**				
Civil status	Married	*Reference*	*Reference*	*Reference*
	Single	1.06 (0.78–1.44)	1.21 (0.80–1.83)	0.97 (0.62–1.52)
ART	No	*Reference*	*Reference*	*Reference*
	Yes	0.88 (0.58–1.31)	0.45 (0.18–1.11)	1.21 (0.76–1.93)
Age	**≤**39	*Reference*		
	**≥**40	1.36 (1.14–1.62)		
**Circulatory (Chapter IX)**				
Civil status	Married	*Reference*	*Reference*	*Reference*
	Single	1.11 (0.86–1.44)	1.14 (0.78–1.67)	1.08 (0.75–1.54)
ART	No	*Reference*	*Reference*	*Reference*
	Yes	0.84 (0.59–1.18)	0.85 (0.45–1.58)	0.81 (0.53–1.23)
Age	**≤**39	*Reference*		
	**≥**40	1.55 (1.34–1.79)		
**Respiratory (Chapter X)**				
Civil status	Married	*Reference*	*Reference*	*Reference*
	Single	1.25 (1.03–1.52)	1.42 (1.11–1.80)	1.05 (0.77–1.45)
ART	No	*Reference*	*Reference*	*Reference*
	Yes	1.20 (0.95–1.52)	1.09 (0.76–1.56)	1.32 (0.97–1.82)
Age	**≤**39	*Reference*		
	**≥**40	0.84 (0.75–0.95)		
**Digestive (Chapter XI)**				
Civil status	Married	*Reference*	*Reference*	*Reference*
	Single	0.98 (0.82–1.17)	0.90 (0.70–1.14)	1.09 (0.84–1.42)
ART	No	*Reference*	*Reference*	*Reference*
	Yes	1.04 (0.84–1.29)	1.09 (0.78–1.50)	1.02 (0.76–1.37)
Age	**≤**39	*Reference*		
	**≥**40	1.00 (0.91–1.11)		
**Skin and subcutaneous tissue (Chapter XII)**				
Civil status	Married	*Reference*	*Reference*	*Reference*
	Single	1.16 (0.97–1.39)	1.02 (0.79.1.32)	1.35 (1.03–1.75)
ART	No	*Reference*	*Reference*	*Reference*
	Yes	1.20 (0.96–1.49)	1.30 (0.94–1.80)	1.13 (0.84–1.52)
Age	**≤**39	*Reference*		
	**≥**40	1.02 (0.91–1.13)		
**Musculoskeletal and connective tissue (Chapter XIII)**				
Civil status	Married	*Reference*	*Reference*	*Reference*
	Single	1.14 (0.96–1.34)	1.06 (0.84–1.34)	1.24 (0.98–1.56)
ART	No	*Reference*	*Reference*	*Reference*
	Yes	1.00 (0.81–1.24)	1.08 (0.78–1.05)	0.99 (0.78–1.30)
Age	**≤**39	*Reference*		
	**≥**40	1.29 (1.18–1.42)		
**Genitourinary (Chapter XIV)**				
Civil status	Married	*Reference*	*Reference*	*Reference*
	Single	1.11 (0.99–1.25)	1.26 (1.08–1.48)	0.94 (0.79–1.12)
ART	No	*Reference*	*Reference*	*Reference*
	Yes	4.85 (4.26–5.53)	8.02 (6.46–9.94)	3.42 (2.88–4.06)
Age	**≤**39	*Reference*		
	**≥**40	1.02 (0.94–1.10)		
Somatic disease*				
Civil status	Married	*Reference*	*Reference*	*Reference*
	Single	1.19 (1.06–1.33)	1.27 (1.09–1.48)	1.09 (0.92–1.28)
ART	No	*Reference*	*Reference*	*Reference*
	Yes	3.18 (2.73–3.70)	5.18 (3.97–6.75)	2.32 (1.92–2.81)
Age	**≤**39	*Reference*		
	**≥**40	1.15 (1.09–1.22)		

Stratifying the data by age, it was found that among the mothers in the control group, being single at time of birth increased the risk for having been diagnosed with *“Infections”* (OR = 1.64, 95% CI = 1.33–2.02), *“Mental and behavioral disorders”* (OR = 1.84, 95% CI = 1.49–2.28), *“Respiratory”* (OR = 1.42, 95% CI = 1.11–1.80), *“Genitourinary”* (OR = 1.26, 95% CI = 1.08–1.48), and *“Any type of somatic disease”* (OR = 1.27, 95% CI = 1.09–1.48), Table 4. The mothers in the control group who had used ART also had an increased likelihood of being diagnosed with *“Neoplasms”* (OR = 1.48, 95% CI = 1.06–1.76), *“Endocrine, nutritional and metabolic”* (OR = 2.97, 95% CI = 2.20–4.03), *“Genitourinary”* (OR = 8.02, 95% CI = 6.46–9.94), and *“Any type of somatic disease”* (OR = 5.18, 95% CI = 3.97–6.75), Table 4. Single mothers age 40 or older had a higher likelihood of being diagnosed with *“Mental and behavioral disorders”* (OR = 1.87, 95% CI = 1.47–2.37), *“Skin and subcutaneous tissue”* (OR = 1.35, 95% = 1.03–1.75) compared to mothers who were cohabiting/married at time of childbirth, while mothers age 40 or older who had undergone ART treatment had an increased likelihood for having been diagnosed with *“Neoplasm”* (OR = 1.35, 95% CI = 1.06–1.73), *“Endocrine, nutritional and metabolic”* (OR = 1.79, 95% CI = 1.36–2.37), *“Genitourinary”* (OR = 3.42, 95% CI = 2.88–4.06), and *“Any type of somatic disease”* (OR = 2.32, 95% CI = 1.92–2.81), Table 4.

In a subgroup analysis of mothers age 45 or older, it was found that mothers who were single at the time of birth had an increased likelihood for being diagnosed with *“Neoplasms”* (OR = 2.75, 95% CI = 1.10–6.88), *“Mental and behavioral disorders”* (OR = 2.67, 95% CI = 1.04–6.86), *“Eye and adnexa”* (OR = 4.45, 95% CI = 1.30–15.30) compared to mothers who were cohabiting/married, while mothers who had used ART had a higher likelihood of being diagnosed with *“Genitourinary”* (OR = 2.17, 95% CI = 1.12–4.18) compared to mothers who had become spontaneously pregnant, data not shown. Also, dividing maternal age at birth in to six categories (>25, 25–29, 30–34. 35–39. 40–44, and > =45) a dose-response relationship was found between maternal age and morbidity due to *“Neoplasms”, “Blood and Immune system”, “Endocrine, nutritional and metabolic”, “Nervous system”, “Eye and adnexa”, “Circulatory”, Musculoskeletal and connective tissue”, and “Somatic disease”* (data not shown) among all women but also in the subgroup of primiparous women only.

### Multivariable analysis of morbidity up to 5 years after childbirth

The overall morbidity, measured by *“Any type of somatic disease”* up to 5 years after childbirth was found to be higher among mothers who had used ART to become pregnant (OR = 2.16, 95% CI = 1.79–2.60) compared to mothers who had become spontaneously pregnant. Also, mothers 40 years or older, who had used ART to become pregnant had an increased likelihood of having been diagnosed with *“Any type of somatic disease”* up to 5 years after childbirth, Table 5. Moreover, mothers, in both age groups, who were single at time of childbirth had an increased likelihood of being diagnosed with *“Infections”* (OR = 1.30, 95% CI = 1.09–1.55), and *“Mental and behavioral disorders”* (OR = 1.50, 95% CI = 1.28–1.75) while mothers age 40 or older also had an increased likelihood of being diagnosed *with “Neoplasms”* (OR = 1.30, 95% CI = 1.18–1.42), *“Eye and adnexa”* (OR = 1.34, 95% CI = 1.20–1.50), *“Ear and mastoid”* (OR = 1.27, 95% CI = 1.10–1.47), *“Circulatory”* (OR = 1.27, 95% CI = 1.10–1.47), *“Skin and subcutaneous tissue”* (OR = 1.12, 95% CI = 1.02–1.23), and *“Musculoskeletal and connective tissue”* (OR = 1.24, 95% CI = 1.12–1.36), Table 5.

**Table 5:** Odds ratios and corresponding 95% confidence intervals (CI) for being diagnosed within each of the presented ICD-10 chapters up to 5 years after childbirth.

			Up to 5 years after childbirth	
		5 years after childbirth	≤39	≥40
		OR (95% CI)	OR (95% CI)	OR (95% CI)
**Infections (Chapter I)**				
Civil status	Married	*Reference*	*Reference*	*Reference*
	Single	1.30 (1.09–1.55)	1.30 (1.03–1.64)	1.29 (0.99–1.69)
ART	No	*Reference*	*Reference*	*Reference*
	Yes	1.06 (0.85–1.33)	0.88 (0.61–1.25)	1.22 (0.92–1.63)
Age	**≤**39	*Reference*		
	**≥**40	0.94 (0.85–1.05)		
**Neoplasms (Chapter II)**				
Civil status	Married	*Reference*	*Reference*	*Reference*
	Single	1.11 (0.94–1.30)	0.86 (0.67–1.12)	1.34 (1.08–1.66)
ART	No	*Reference*	*Reference*	*Reference*
	Yes	1.11 (0.96–1.37)	1.04 (0.77–1.39)	1.22 (0.97–1.52)
Age	**≤**39	*Reference*		
	**≥**40	1.30 (1.18–1.42)		
**Blood and Immune system (Chapter III)**				
Civil status	Married	*Reference*	*Reference*	*Reference*
	Single	0.99 (0.74–1.34)	0.94 (0.62–1.42)	1.04 (0.67–1.62)
ART	No	*Reference*	*Reference*	*Reference*
	Yes	0.95 (0.67–1.35)	0.65 (0.34–1.21)	1.17 (0.76–1.81)
Age	**≤**39	*Reference*		
	**≥**40	1.09 (0.92–1.29)		
**Endocrine, nutritional and metabolic (Chapter IV)**				
Civil status	Married	*Reference*	*Reference*	*Reference*
	Single	1.04 (0.87–1.24)	1.16 (0.92–1.46)	0.86 (0.64–1.13)
ART	No	*Reference*	*Reference*	*Reference*
	Yes	1.18 (0.96–1.45)	1.21 (0.88–1.66)	1.11 (0.84–1.46)
Age	**≤**39	*Reference*		
	**≥**40	0.91 (0.82–1.01)		
**Mental and behavioral disorders (Chapter V)**				
Civil status	Married	*Reference*	*Reference*	*Reference*
	Single	1.50 (1.28–1.75)	1.49 (1.21–1.82)	1.52 (1.19–1.93)
ART	No	*Reference*	*Reference*	*Reference*
	Yes	0.86 (0.68–1.08)	0.84 (0.59–1.20)	0.88 (0.64–1.21)
Age	**≤**39	*Reference*		
	**≥**40	0.98 (0.89–1.09)		
**Nervous system (Chapter VI)**				
Civil status	Married	*Reference*	*Reference*	*Reference*
	Single	0.93 (0.75–1.15)	0.89 (0.66–1.20)	0.99 (0.73–1.36)
ART	No	*Reference*	*Reference*	*Reference*
	Yes	1.08 (0.85–1.38)	0.97 (0.66–1.42)	1.26 (0.92–1.72)
Age	**≤**39	*Reference*		
	**≥**40	1.08 (0.96–1.22)		
**Eye and adnexa (Chapter VII)**				
Civil status	Married	*Reference*	*Reference*	*Reference*
	Single	1.08 (0.88–1.32)	0.92 (0.67–1.26)	1.22 (0.94–1.59)
ART	No	*Reference*	*Reference*	*Reference*
	Yes	1.31 (1.06–1.62)	1.16 (0.81–1.66)	1.41 (1.08–1.83)
Age	**≤**39	*Reference*		
	**≥**40	1.34 (1.20–1.50)		
**Ear and mastoid (Chapter VIII)**				
Civil status	Married	*Reference*	*Reference*	*Reference*
	Single	0.94 (0.72–1.23)	1.06 (0.73–1.53)	0.85 (0.57–1.26)
ART	No	*Reference*	*Reference*	*Reference*
	Yes	0.92 (0.79–1.12)	0.78 (0.47–1.30)	1.06 (0.72–1.56)
Age	**≤**39	*Reference*		
	**≥**40	1.27 (1.10–1.47)		
**Circulatory (Chapter IX)**				
Civil status	Married	*Reference*	*Reference*	*Reference*
	Single	1.08 (0.90–1.32)	1.07 (0.80–1.43)	1.12 (0.85–1.47)
ART	No	*Reference*	*Reference*	*Reference*
	Yes	0.85 (0.66–1.09)	0.77 (0.50–1.19)	0.91 (0.66–1.24)
Age	**≤**39	*Reference*		
	**≥**40	1.27 (1.10–1.47)		
**Respiratory (Chapter X)**				
Civil status	Married	*Reference*	*Reference*	*Reference*
	Single	1.02 (0.86–1.22)	1.13 (0.89–1.43)	0.90 (0.68–1.18)
ART	No	*Reference*	*Reference*	*Reference*
	Yes	1.19 (0.98–1.45)	0.96 (0.70–1.33)	1.34 (1.04–1.73)
Age	**≤**39	*Reference*		
	**≥**40	1.01 (0.91–1.12)		
**Digestive (Chapter XI)**				
Civil status	Married	*Reference*	*Reference*	*Reference*
	Single	1.02 (0.87–1.19)	1.10 (0.90–1.35)	0.91 (0.72–1.16)
ART	No	*Reference*	*Reference*	*Reference*
	Yes	0.93 (0.77–1.12)	0.92 (0.68–1.24)	0.91 (0.70–1.17)
Age	**≤**39	*Reference*		
	**≥**40	1.05 (0.96–1.15)		
**Skin and subcutaneous tissue (Chapter XII)**				
Civil status	Married	*Reference*	*Reference*	*Reference*
	Single	1.04 (0.88–1.23)	0.88 (0.69–1.13)	1.24 (0.98–1.56)
ART	No	*Reference*	*Reference*	*Reference*
_	Yes	1.07 (0.89–1.30)	0.97 (0.72–1.32)	1.15 (0.90–1.47)
Age	**≤**39	*Reference*		
	**≥**40	1.12 (1.02–1.23)		
**Musculoskeletal and connective tissue (Chapter XIII)**				
Civil status	Married	*Reference*	*Reference*	*Reference*
	Single	1.06 (0.90–1.26)	0.95 (0.75–1.21)	1.22 (0.96–1.56)
ART	No	*Reference*	*Reference*	*Reference*
	Yes	0.67 (0.54–0.82)	0.63 (0.45–0.88)	0.72 (0.55–0.95)
Age	**≤**39	*Reference*		
	**≥**40	1.24 (1.12–1.36)		
**Genitourinary (Chapter XIV)**				
Civil status	Married	*Reference*	*Reference*	*Reference*
	Single	0.95 (0.83–1.08)	1.06 (0.89–1.28)	0.82 (0.67–1.00)
ART	No	*Reference*	*Reference*	*Reference*
	Yes	3.72 (3.17–4.37)	5.38 (4.14–6.99)	2.86 (2.33–3.51)
Age	**≤**39	*Reference*		
	**≥**40	1.06 (0.98–1.14)		
Somatic disease*				
Civil status	Married	*Reference*	*Reference*	*Reference*
	Single	1.10 (0.96–1.25)	1.11 (0.93–1.33)	1.07 (0.88–1.29)
ART	No	*Reference*	*Reference*	*Reference*
	Yes	2.16 (1.79–2.60)	2.44 (1.81–3.30)	1.95 (1.54–2.48)
Age	**≤**39	*Reference*		
	**≥**40	1.12 (1.05–1.21)		

Stratifying by age, it was found that younger mothers who were single at time of birth had an increased likelihood of being diagnosed with *“Infections”* (OR = 1.30, 95% CI = 1.03–1.64) and *“Mental and behavioral disorders”* (OR = 1.49, 95% CI = 1.21–1.82), Table 5. Mothers in this age group who had used ART had an increased likelihood of having been diagnosed with *“Genitourinary”* (OR = 5.38, 95% CI = 4.14–6.99), while having a decreased likelihood of being diagnosed with *“Musculoskeletal and connective tissue”* (OR = 0.63, 95% CI = 0.45–0.88). Single mothers in the index group had a higher likelihood of being diagnosed for *“Neoplasms”* (OR = 1.34, 95% CI = 1.08–1.66) and *“Mental and behavioral disorders”* (OR = 1.52, 95% CI = 1.19–1.93) compared to women who were cohabiting/married at time of childbirth, Table 5. Moreover, mothers in this age group (≥40 years of age) who had used ART to achieve pregnancy had a higher likelihood of being diagnosed with *“Eye and Adnexa”* (OR = 1.41, 95% CI = 1.08–1.83), *“Genitourinary”* (OR = 2.86, 95% CI = 2.33–3.51), *“Respiratory”* (OR = 1.34, 95% CI = 1.04–1.73) and *“Any type of somatic disease”* (OR = 1.95, 95% CI = 1.54–2.48) and a decreased likelihood for being diagnosed with *“Musculoskeletal and connective tissue”* (OR = 0.72, 95% CI = 0.55–0.95), Table 5.

As for morbidity prior to childbirth, dividing maternal age at birth in to the same six categories, a dose-response relationship was found between maternal age and morbidity due to *“Neoplasms”, “Blood and Immune system”, “Nervous system”, “Eye and adnexa”, “Circulatory, and “Somatic disease”* among all women as well as in the subgroup of primiparous women only (data not shown).

## Discussion

In this case-control /observational epidemiological study of women’s morbidity five years before and up to five years after childbirth, we found that spontaneously pregnant mothers who were 40 years of age or older at time of childbirth were more likely to have been diagnosed for several diagnosis chapters in ICD-10, in specialized care, both during the five years prior to and the five years following childbirth compared to their younger counterparts. Diagnosis within *“Neoplasms”, “Blood and Immune system”, “Endocrine, nutritional and metabolic”, “Mental and behavioral disorders”, “Ear and Mastoid”, “Circulatory”, “Musculoskeletal and connective tissue”* and *“Genitourinary”* were more common in the older age group.

Advanced maternal age as an independent variable for a range of pregnancy and delivery-related complications and as a risk factor for worse health postpartum as has been reported in previous studies^10–13^. Our results confirm that age is a risk factor for higher morbidity. The current study also found a dose-response relationship between maternal age and morbidity prior and after childbirth.

We also found that mothers in both age groups (≤39 and ≥40 years of age), who had achieved pregnancy by using assisted reproductive technology (ART), were more than twice as likely to have been diagnosed with *“Any type of somatic disease”* during the five years following childbirth compared to spontaneously pregnant women. Also, up to five years before childbirth, the mothers using ART had been significantly more often diagnosed with *“Neoplasms”, “Endocrine, nutritional and metabolic disorders”* and more than three times more likely to have been diagnosed with *“Any type of somatic disease”* compared to mothers spontaneously pregnant.

Previous studies have concluded that there are higher overall risks for negative maternal and neonatal outcomes for conception resulting from use of ART compared to spontaneous conceptions^3,31–34^ and our result - higher morbidity both before and after childbirth, in women using ART- further indicates the need for giving greater attention to this group and doing further research on the use of ART for women with comorbidity to infertility.

The older mothers were more often single, regardless of type of pregnancy, compared to the younger mothers and ART-treated mothers were more than twice as often single. Divorce, separation or partner’s death are probable explanations for the mothers’ single status. However, single mothers pursuing parenthood on their own, both by usage of ART and by natural conception are probably also present in the group of single mothers. This is in line with previous studies describing women who become planned single mothers (also referred to as solo mothers) tending to be of higher age than partnered women^20,35^.

Substantially worse birth outcomes for single women than for cohabiting or married women have been reported^26,36^. Our results, showing that older single mothers giving birth have a significantly higher morbidity compared to both married/cohabiting mothers of the same age and compared to younger mothers whether single or married respectively probably indicate that being single is a factor contributing to increased risk of adverse birth outcomes.

This as well as the overall worse health amongst ART-using single and married women, calls for giving more attention to the ever-increasing number of single women pursuing parenthood by planning to become single mothers.

Of specific interest is our result that mothers in both age groups and regardless of type of pregnancy, who are single at time of childbirth, had an increased likelihood of being diagnosed with mental and behavioral disorders both up to five years before as well as up to five years after childbirth compared to cohabiting or married mothers. According to previous studies, the lack of a partner is one of many factors associated with psychiatric illness during and after pregnancy^37–40^. Parental mental health problems are highly negatively correlated with children’s assessment of their own health and children growing up in single mother families are at larger risk for emotional problems^41^. The Fragile Family Study in the United States found that children growing up with single mothers are at elevated risk of poorer health^23^ and the Millennium Cohort Study in the United Kingdom found that children of single mothers had more psychological problems. This was associated with the mother’s worse health and her economic disadvantage compared to the health and financial situation of partnered parents^42^. Single status is considered to be a psychosocial risk factor within antenatal care today.

The women who chose to become single mothers were not specifically identified in this study, so we do not know if the poorer health seen in single mothers in this study would also be seen in mothers who had become single unintentionally. The active choice of becoming a single mother is sometimes seen as being protective against the psychological distress often considered to be generated from unplanned pregnancy or single status due to divorce or separation. However, a recent study by Munk-Olsen *et al*.^14^ showed that even the women with a planned IVF pregnancy had a significantly heightened risk of experiencing psychiatric episodes requiring treatment at a psychiatric facility after delivery compared to women not giving birth. This is of interest in our discussion since the planned single mothers who used ART (IVF with donated sperm) sometimes are excluded from researchers’ discussions of the risks of single motherhood. Perhaps pregnancy intention does not reduce the risk of psychiatric illness and, as recently noted, single mothers with psychiatric illness are far more vulnerable and their psychiatric illness increases the risk that the child’s health will be adversely affected.

Women who plan to be single mothers, are sometimes described as being primarily professional women with higher education and more financially secure and therefore better protected against physical and mental health problems faced by mothers to be who are less well off. These women are also more likely to be able to afford using ART. In Sweden and a few other countries, ART is mainly financed by the government, a fact that consequently might not call for these otherwise protective characteristics in the single mother by choice, as when ART is privately financed. It is generally easier to become a single parent in Sweden and some other countries today than it was a few decades ago. If this leads to a growth in the size of the single-parent-by choice group, further research on this group is needed. However, the results of our study indicate that health care needs to give more attention to the ever-growing population of single mothers, not just to any special subgroup (21,39,41).

There has been a global increase in the percentage of children who grow up in single-parent households which suggests that more attention needs to be given to this group in many countries^43^. However, in Sweden, it has actually become less common for children to grow up in single-parent households^44^. The risk associated to single parenthood for both mother and child^23,45,46^ calls for interventions aiming to promote the well-being of the mothers and by extension improving child adjustment. In a recent study, Taylor and Conger discuss how to promote strength and resilience in single mothers by considering factors amenable to change via behavioral interventions, for example by providing peer-group support combined with cognitive-behavioral training^47^.

Through multivariable statistical analysis we found that single status, pregnancy by use of ART and being over 40 years of age at time of childbirth, respectively, increased the likelihood of having been diagnosed with *“Any type of somatic disease”* (i.e. diagnosis in chapter 1–14 in ICD-10) during the five years prior to childbirth, while after childbirth only the use of ART and being 40 years of age or older entailed an increased risk of having been diagnosed with *“Any type of somatic disease”*. The older mothers were more often single compared to the younger mothers; they had more often used ART to become pregnant and had more often a chronic disease and a higher morbidity. It appears that more and more women will choose postponement of parenthood suggesting that we are most likely to see an increase in the percentage of older single women with health problems who choose to use ART. This suggests that healthcare providers must look forward to a growing need for care for this population.

Maternal single status is associated with an increased risk for giving birth to a child with low birth weight, small for gestational age and pre-term^26^. The present study shows that the older mothers had a higher number of diagnoses and were more often single than their younger counterparts. Women (and men) postpone having children to a later time in life due to improved social conditions, better contraceptives and time-consuming academic and work-related achievements. Also, an increased societal acceptance of postponing parenthood to a later time in life, meeting a partner later in life, and a wish to establish financial security before starting a family^3,10^ contribute to the postponement of parenthood. The underestimation of the impact of age-related factors on female fertility and the overestimation of the use of ART in overcoming age-related infertility^48–53^ probably contribute to the postponement of childbearing. The substantial risks for both the woman and the child associated with giving birth at advanced maternal age^21,39,54^ call for appropriate physician counseling of older women who are considering ART^55^. It appears that education about fertility and infertility should be given a more prominent place in health programs focused on how to prevent sexually transmitted diseases and unwanted pregnancies in schools and health settings.

Previous studies of mother’s mental and physical health and morbidity, before and after childbirth, are to a large extent based on self-reported questionnaires or interviews analyzed with qualitative methods. Although problems have been reported after childbirth, mother’s self-rated quality of life tend to differ due to mode of delivery and civil status and most of them perceive their health as good^16,17,56^. The current study contributes to building a body of knowledge about older mothers in a more objective manner than is possible with self-report questionnaire studies. The main results in our study are based on the diagnoses reported by physicians at in- and outpatient specialized care units who have clinically examined older mothers. Primary care data and findings from visits not involving a physician are not entered in the registers from which our data were collected, which further strengthen the validity of this study. The symptoms leading to a specialized care visit are also assumingly more severe and the diagnoses registered are thus more serious.

The large number of mothers included in this study is a major strength because it reduces the importance of a range of error sources such as skewness in recruitment and participation. The underreporting of inpatient data to the National patient register is estimated to be less than 1%. This validates our study’s findings even further.

Definite conclusions about causality cannot be made due to the study’s observational design but the study does contribute to the knowledge of the role of maternal age, civil status and use of ART in affecting a woman’s morbidity. In accordance with previous knowledge of advanced age-related and single status-related risks for worse outcome for mothers, our findings further enhance the need for careful considerations in planning and providing reproductive guidance for older women. Being fully informed about risks that may affect the future mental and physical health of both the woman and child is advisable. Age is obviously a factor contributing to higher morbidity in the group of mothers in our study, and probably provides part of the explanation for higher morbidity after childbirth as well. However, the vulnerability to both physical and mental strains that characterizes the mere pregnancy and postpartum-period, in combination with pre-existing medical conditions, could, in part explain our results and this is in line with a recent study by Morris *et al*.^57^.

## Conclusions

The increased risk of several somatic and mental and behavioral disorders amongst older, single, or ART treated mothers points to the importance of developing new policy outlining a long-term commitment to developing systems to inform the women and to carefully consider interventions and treatments needed by an ever-increasing group of women who become pregnant at what is considered to be an advanced age. More resources need to be provided to support preventive actions and the provision of evidence-based information on fertility and reproduction for younger women at all societal levels with one goal being to reduce the number of pregnancies in women of advanced age.
